# A Novel Clinically Immersive Pre-doctoral Training Program for Engineering in Surgery and Intervention: Initial Realization and Preliminary Results

**DOI:** 10.1007/s43683-021-00051-2

**Published:** 2021-06-07

**Authors:** Michael I. Miga, Robert F. Labadie

**Affiliations:** 1 Department of Biomedical Engineering, School of Engineering, Vanderbilt University, Nashville 37235, USA;; 2 Vanderbilt Institute for Surgery and Engineering, School of Engineering, Vanderbilt University, Nashville 37235, USA;; 3 Department of Radiology and Radiological Sciences, Vanderbilt University Medical Center, Nashville 37232, USA;; 4 Department of Neurological Surgery, Vanderbilt University Medical Center, Nashville 37232, USA;; 5 Department of Otolaryngology–Head and Neck Surgery, Vanderbilt University Medical Center, Nashville 37232, USA

**Keywords:** Biomedical engineering, Experiential learning, Graduate education, Immersion, Surgery, Intervention

## Abstract

A novel pre-doctoral program is presented that combines (1) immersive observation in the surgical/interventional theatre and (2) thought-provoking exposition activities focused on answering clinically provocative questions. While the long-term goal is to train engineers to conduct clinical translational research in human systems, in this paper, perceived trainee improvements are assessed in: (1) their ability to pose important questions in surgery and intervention, (2) their knowledge of surgical technologies, and (3) their understanding of procedural medicine. The program combines constructivist and constructionist learning approaches through a dual-course suite consisting of: (1) a scaffold lecture design with ten physicians presenting their procedural specialties interleaved with lectures relating engineering principles, and (2) a second course with clinically mentored immersion experiences in the operating room/interventional suite, clinical conferences, and patient rounds. Details of the complementing technical core and learning environment are also provided. Preliminary data reports on the quantitative experiential clinical involvement and on a self-reported survey over 5 cohorts of trainees (*n* = 18). With respect to immersion, the average surgeries/interventions observed, number of different types, and clinical contact time per student was on average 15.6 ± 7.9 surgeries/interventions, 8.2 ± 3.6 types, and 48.2 ± 14.7 contact hours, respectively. With respect to trainee understanding of procedural medicine, surgical technologies, and value of clinical observation, an average perceived improvement of 41%, 38%, and 41% over the course series was detected, respectively (*p* < 0.001). Equally impressive, when rating ability to pose important questions affecting human health, an average perceived improvement of 34% was detected (*p* < 0.001). The preliminary realization of a novel pre-doctoral clinically immersive training program for engineering trainees is described and demonstrates extensive levels of clinical contact and strong evidence that the provided immersion experiences result in significant improvements in understanding of procedural medicine.

## INTRODUCTION

Experiential learning with a focus on clinical immersion, albeit sparse, is a growing trend in the biomedical engineering community with early programs focused on medical device design such as Stanford Byers Center for Biodesign.^1^ While the Stanford program is primarily a postgraduate experience, other institutions have focused on undergraduate and graduate students. For example, at Johns Hopkins University (JHU), undergraduate biomedical engineering students participate in a 2 week clinical immersion experience in preparation for their senior design projects.^2^ Others offer summer experiences and/or team immersion approaches that mix engineering and medical students in the hopes of leveraging different perspectives in identifying and solving clinically applicable problems.^3–5^ An excellent review of these larger undergraduate efforts can be found in Reference^6^. Clinical immersion for traditional graduate engineering education is much less common, perhaps due to a lack of integration among clinical and engineering program infrastructure as suggested by Lee and Jabloner.^7^ Unfortunately, without these experiences, engineering research risks not providing an appreciation of the broader clinical problem by narrowly focusing on engineering methods and approach. In a separate effort addressing this, JHU has developed a 1 year master’s program where students begin with a two-stage clinical immersion framework.^8^ Stage 1 consists of four clinical rotations (2 core and 2 elective) including surgical observation, grand rounds, shadowing clinicians in both inpatient and outpatient setting, and interviewing clinical staff. Following stage 1, which occurs in a state-of-the art facility, stage 2 consists of clinical immersion in a low resource environment. Quantitative results on the nature and depth of clinical exposure in the program are not provided; however, an impressive degree of attracted design project funding is reported as well as invention disclosures and patent applications.

A common thread to the programs described above is that the clinical immersions are largely framed as an exposure to standard-of-care procedural medicine with the intent of utilizing engineering technology to improve and innovate. While laudable, such programs are not structured for hypothesis-driven, clinical-translational research. In an effort to address, a pre-doctoral engineering training program has been designed that employs the learning approaches of constructivism^9^ and constructionism^10^ within the context of fostering novel research in the domains of surgery and intervention. Briefly, constructivism states that knowledge is generated by the experience itself and is not overly concerned with formal context or tools of delivery. Here, learning is achieved indirectly from unstructured experiences of the surrounding environment.^11^ In contrast, constructionism promotes situated learning and context and subscribes to the projection of that knowledge in the form of substantive work or a “public entity” that shapes and molds ideas through communication.^11^ Using these learning approaches, the training program’s basic premise reported here subscribes to a wider precept designed to connect engineers with the clinical cadre in an institutional community building program. The program advocates that properly trained engineers could play a pivotal role for the realization of transformative multi-disciplinary teams. While in the past, clinician–scientists would serve this role, in recent years the discipline’s population has been declining.^12^ For example, according to the American Medical Association, during an 18 year period ending in 2003, the number of physicians involved with research decreased by 60% while the number of physicians involved in patient care doubled.^13^ More recently, this decline has slowed (from 2003 to 2012, the decrease in physicians involved in research saw a 17% drop), but younger physicians (age cohort 31–60) still reflected a 30% decrease on average.^14^ While the most significant contributing causes to the decline are a subject of debate (e.g. increasing cost of medical education, increasing length of clinical training, physician–scientist being increasingly asked to support higher percentages of salary by treating patients, *etc*.), its occurrence is widely recognized and as Salata *et al*. states, it “renders the system vulnerable to collapsing”.^12^ As a result, when considering the increasing complexity of research, the changing nature of healthcare teams, the practical stressors associated with the physician workforce, and the need for institutional-wide integrated support, appropriately trained engineers could serve a critical role in addressing this gap and in accelerating innovative therapeutic approaches and/or facilitating the study of human disease. The program presented herein was created to address this role by combining advanced engineering knowledge with a comprehensive clinical immersion in procedural medicine. The initial realization with preliminary assessment data is reported here.

## PROGRAM DESCRIPTION

### Learning Approach

The overarching learning theory associated with this training program is based on the experiential learning model expressed in the work by Ackermann.^11^ In Reference^11^, Ackermann describes the distinct benefits from creating a learning environment that combines the concepts of Piaget’s constructivism^9^ and Papert’s constructionism.^10^ In Piaget’s model, learners often have preconceived views and potentially resist changes despite the agitation of new data from experiences. Piaget advocates that the immersion of the trainee into the environment, while potentially generating a loss of order, leads to the necessary generation of a learning framework, i.e. the process of creating structure from disorder enables learning. In contrast, Papert supports more structured experiences and often an iterative self-directed learning whereby students generate their own tools to support understanding and the development of knowledge.^11^ It is clear that both have important supportive contributions to learning.

While Papert’s direction builds on Piaget’s model by suggesting a certain efficiency associated with taking a step back to provide context, there are commonalities between the two. Both theories believe in the incremental introduction of knowledge as a source of conceptual change. Both believe that improved learning is codified in a process that balances constancy and adjustment. And, both believe in a scholar’s ability to adapt as an important component to learning.^11^ However, one important difference concerns the frame of reference of the learner. Piaget advocates for a complete immersion into the experience to optimize learning, i.e. a relative reference where the scholar is the reference frame and knowledge is gained by the engaged environment. In contrast, Papert advocates for a fixed reference of context for the events that are taking place. Ackermann’s contribution assimilates the views stating that “…distancing oneself from a situation does not necessarily entail disengaging, but may constitute a necessary step toward relating even more intimately and sensitively to people and things. In any situation, it would seem, there are moments when we need to project part of our experience outwards, to detach from it, to encapsulate it, and then to reengage with it. This view of separateness can be seen as a provisory means of gaining closer relatedness and understanding.^11^“ This integrated view is an underlying characteristic of the program reported in this work. More specifically, students are gradually introduced to a learning framework that translates its emphasis between Papert’s and Piaget’s model via two sequential clinical immersion courses.

Briefly explained, training begins with a course titled *Engineering in Surgery and Intervention: Provocative Questions* (*ESI:PQ*) which is context heavy and consists of physicians introducing their specialty and conveying their experiences in the operating room/interventional suite. During the course, physicians provide a variety of provocative questions to the students focusing on significant clinical barriers or missing fundamental disease/therapeutic understanding that could be potentially transformative if resolved. Predominantly, *ESI:PQ* reflects a structured learning environment. Lectures are often presented by the physician in a somewhat didactic manner as to what the goal is wanting to be achieved and what barrier or lack of understanding is inhibiting its achievement. Concepts from engineering and medicine are blended in interleaving technical lectures from the non-clinical instructor to highlight capabilities that could possibly bear on a problem or perhaps studies that could be conducted to shed light on missing knowledge. Examples are often pulled from the literature to demonstrate the current state-of-the-art. As will be discussed below, systematic expository deliverables are requested of the student which fulfill Papert’s constructionism approach that requires the projection of knowledge. It should also be noted however that the *ESI:PQ* course is not divorced of Piaget’s constructivist influence. One could certainly argue that the relaying of procedural videos in our clinical sessions within the course have the hallmark of Piaget’s model. More specifically, as a physician relays a complex cascade of surgical/interventional steps relaying treatment within a video, image sequence, or flowchart of care, trainees are certainly provided a preview of a somewhat unguided immersive environment in terminology, data, and visual experiences that are quite foreign and unstructured. These moments certainly provide impression while also promoting disorder to encourage creating understanding.

In the second course, titled *Engineering in Surgery and Intervention: Clinical Interactions* (*ESI:CI*), an intensive immersive environment is introduced to the students by embedding them within a clinical team with little didactic interaction. Piaget’s constructivism model associated with unstructured immersion is certainly heavily supported in this experience. The direct observation of the surgical and interventional field, to include the experience of the sterile dynamic as well as preoperative preparation, done with essentially little guidance other than to not invade the sterile field and always be situationally aware of ‘scrubbed-in’ participants is certainly compatible with the disordered immersive framework of the constructivist. In addition, a second similar experience, although not quite as disordered, is attending clinical conferences. In these discussions, trainees witness the trajectory of care for individual patients being developed. These patient plans may involve three and four specialties all working in concert to decide the best course of action. While there is structure to these presentations, the sheer extent of data and presentation information being used is certainly a somewhat disordered experience for the trainee, again fitting with Piaget’s model. Similar to the first course, *ESI:CI* still injects content from the other learning model as well. In this case, after each weekly experience, classroom activity revolves around a task that requires the students to “report out” their experiences and discussion ensues to add context to disordered experiences. Major expository assignments are periodically requested as well.

With respect to the proposed learning approach, the overall long-term programmatic hypothesis is that they support a constant cognitive interaction between immersive observation and thought-provoking exposition on clinical questions, and that engineers so trained, will be better able to conduct research in human systems and accelerate the process of translation. In the immediate short-term, the premise of this paper is that this paradigm will lead trainees to improve: (1) their ability to pose provocative questions in surgery and intervention, (2) their knowledge of surgical technologies, and (3) their understanding of what is entailed in clinical translation research. Proving our long-term hypothesis will require extensive internal program evaluation across cohorts as well as long-term outcome analysis of trainee career trajectory. This level of analysis is outside the scope of this initial program realization at this time.

### Program Overview

The training program developed is a multi-year program with candidates being identified in their first year of graduate study and joining the training program at the conclusion of year 1. With respect to participant backgrounds, trainees in the program are from biomedical engineering, electrical engineering, mechanical engineering, and computer science. Selection to trainee status is based on a variety of factors to include academic performance in year 1, as well as research and educational statements/interests, and support from faculty investigators in a domain of procedural medicine. The training program itself consists of a professional development course, the clinical immersion two-course suite, and a broad didactic technical core, when all combined serve to address the professional, clinical, and engineering needs of the trainees. Students also participate in a dynamic seminar series with invited thought leaders, and a separate summer instructional seminar series that highlights Research-in-Progress seminars (RiPs) and the mentoring of undergraduates. In addition, trainees are required to create Individual Development Plans (IDP) reviewed annually to aid in recognizing their own progress. After the training program is completed, students are encouraged to pursue their own projects funded by pre-doctoral fellowship grants (e.g. NIH-F31, NSF trainee award) although the majority are supported by their mentor’s funding.

**FIGURE 1.**
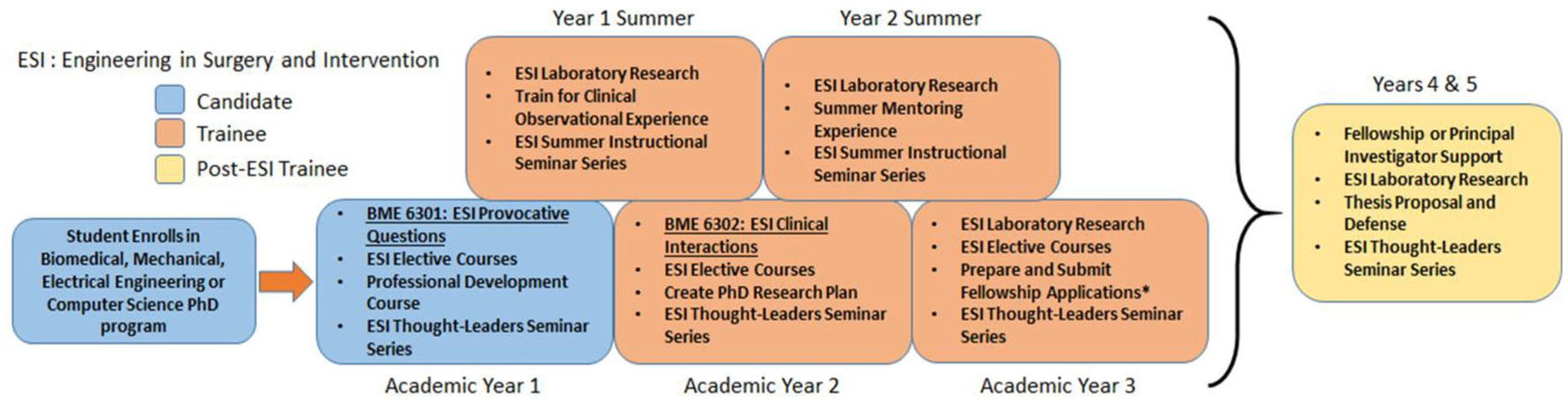
Multi-year *ESI* training framework.

Figure 1 shows the structure of the program. A novel feature of the program is the early exposure of trainees to the clinical domain (second semester of their first year). Thematically, this is an important design feature and is referred to in the pedagogy as ‘real domain experiences’. Too often, rather than beginning with the observations of clinical barriers with subsequent novel ideation, graduate students are influenced by well characterized pre-existing laboratory frameworks. As alluded to in the *Learning Approach*, this habitual indoctrination could inhibit the training of the student as an investigator and could potentially stifle innovation. To ameliorate this effect, interested students enroll in their first year in the first clinical immersion course—*ESI:PQ* (labeled BME 6301 in Fig. 1). Within this first experience, students are exposed to the practice of procedural medicine from as many as 10 surgical and interventional specialties. *ESI:PQ* is then followed by a second even more clinically immersive course that involves one-on-one contact in a medical specialty domain—*ESI:CI* (labeled BME 6302 in Fig. 1). In addition to this clinical suite, a dynamic technical elective core in *ESI* is also taken that allows students to effectively integrate both technical and clinical domains seamlessly at an early stage before they have had extensive exposure to a laboratory environment. With respect to the *ESI* technical core, it is largely reflective of the research themes of participating faculty and is described below (Section Training Core, Subsection ESI: Technical Core).

### Faculty Component

To support this programmatic endeavor, two teaching cadre—(1) engineering/clinician–scientist mentors, and (2) procedural surgical/medicine educators—work synergistically. The engineering/clinical-scientist mentors are comprised of extramurally funded researchers (e.g. NIH R01 investigators) with a primary role of providing a supportive laboratory setting and resources. The procedural surgical/medicine educators are typically involved with research and often are co-investigators on clinically relevant projects in collaboration with the engineering school. Their role is to facilitate the clinical immersion experience and to solidify trainee knowledge with respect to clinical significance and impact.

### Training Core

#### ESI: Clinical Immersion Core

The operating theatre and interventional suites are a rich source of innovative research and educational opportunities. Too often, translating a therapeutic is seen as an end-product development, is undervalued in terms of scientific value, and is often poorly realized such that important potential findings in human systems are confounded. To address these deficits, this program represents an immersion approach with trainees experiencing the clinical environment in a concentrated dual-course core targeted at: (1) understanding surgical/interventional state-of-the-art practices, (2) developing research needs assessments, (3) proposing clinically feasible approaches to breaking down treatment and therapy barriers, and (4) proposing provocative questions for new investigations. Going further, rather than reinforcing contention between basic science and translational work,^7^ emphasis in this program is placed on the concept that translation is an intrinsic part of the scientific method. Moreover, in our experience, there are aspects to human disease and dysfunction that can only be illuminated by translational work and, as such, provide feedback to basic science pursuits. The clinical core subscribes to the tenet that translation is not considered just the work that happens ‘after the science is complete’ but rather, is integral to the science. In this vein, the surgical/interventional platform technologies created, while enabling treatment, also provide opportunities for measurement and discovery within human disease/dysfunction systems. The clinical immersive courses created to achieve these outcomes are described below.

(a) *BME 6301 Engineering in Surgery and Intervention: Provocative Questions*: *ESI:PQ* is a semester-long 3 credit-hour first course in a dual-course suite designed to provide in-depth clinical immersion for trainees. The course is a scaffold design where ten or more physicians from the clinical cadre provide medical specialty lectures throughout the term accompanied by additional didactic lectures relating procedural medicine topics to basic engineering principles typically associated with therapy delivery. Each clinical lecture begins with a discussion of disease background, and the most common and challenging procedures/interventions/treatments in the practice of their procedural art. In the latter half of the lecture, the physician poses provocative questions, and discussion inevitably ensues with the students. To help guide the physicians with respect to *provocative question* development, the following directive is provided, ‘Can you provide a *provocative question* in your area of procedural medicine such that if resolved could dramatically impact treatment efficacy or existing barriers, shift clinical paradigms or training, or fundamentally advance the understanding of disease or therapy? Inspiring provocative questions are questions that encourage creativity and lateral thinking. Good questions inspire analysis, synthesis, interpretation, new experimental methods, and critical thinking. Provocative questions reveal perceived constraints, encourage probing of their veracity, and promote solutions toward significant clinical impact.’ A few examples of provocative questions in past course offerings were: (1) ‘What measurement tools could be applied to chemoembolization to know that a satisfactory embolization endpoint has been reached in real time?’, (2) ‘Clinical trials have shown that an abscopal effect sometimes occurs when radiation is given to patients with solid metastatic cancer who are also on immunotherapy. Why do some patients receive an abscopal effect while others do not?’, or (3) ‘Is there a means to non-invasively determine if a patient’s optic nerve has the potential for neuro-recovery?’

With respect to trainee evaluation, three written practicals are assigned throughout the course consisting of a grouping of the provocative questions from 3 to 4 physicians’ talks. The trainees are asked to select one provocative question for the topic of their practical with guidelines for their written product to be a concise exposition focused upon demonstrating understanding of disease, discussing existing therapeutics and their treatment mechanisms, and then creating a proposed solution which includes hypothesis, supporting specific aims, significance of problem and its resolution. The resolution is a general methodological overview of an approach which includes a brief study design. It should also be noted that trainees are informed to propose projects that are appropriate in scale (2–3 years) and that solutions to provocative questions may be too extensive for this concise scale. If this is the case, they are encouraged to research the problem and come up with appropriately scaled aspects aimed at addressing the provocative question through investigation. In addition to the practicals, engineering exercises designed to illuminate therapeutic mechanisms are assigned for homework on a regular basis as well.

(b) *BME 6302 ESI: Clinical Interactions*: *ESI:CI* is also a semester-long 3 credit-hour course. In this course, trainees experience an in-depth immersion of the clinical theatre, the workflow, and the execution of procedural medicine as well as the surgical/interventional technologies currently employed. Based on trainee interests, a mentor among the clinical cadre is identified and a mentor-trainee agreement is formally made with explicit expectations on each side (e.g. the trainee will arrive on-time and dressed appropriately for interactions and the mentor will provide access to their schedule and a preferred method of communication with the trainee). Clinical experiences involve visits to the operating room/interventional suite, one-on-one consultation with the clinical mentor, attendance at clinical conferences to understand the trajectory of care, and observations of clinical rounds. The premise of this training is that it is too easy to create technology platforms within the laboratory that have an inability, in a realistic time window, to be clinically translated and achieve efficacy. In addition, with increased importance in understanding outcomes in the age of data science, procedural medicine will be influenced and will likely require rigorous quantitative capturing of the trajectory of care to determine best clinical practices. Without an intimate understanding of standard of care and practical workflows within surgical and interventional suites, the barriers to creating technologies that both treat and serve the need for outcome assessment become even more challenging. In addition to clinical observations, in-classroom activities are conducted with interactive assignments including understanding review papers, research proposal writing, clinical barrier identification, and an introspective activity called ‘Reflections’. Reflections are shared each week of the 14 week course among the group of 4–5 students with both an engineering faculty member as a well as a clinical faculty member present. Experiences from the operating rooms and interventional suites are discussed, and students suggest their own provocative questions based on their observations. Faculty members contribute to the discussion by providing background knowledge on technologies used, unusual workflows witnessed, or the nature of clinical findings. These background discussions provide trainees with an “inquiry safe zone” that allow the answering of questions that perhaps could not be asked in an urgent clinical observational setting. Lastly, the evaluation of trainees in this second course include the generation of a comprehensive review paper with guidance from their clinical mentor using techniques in *narrative synthesis*,^15^ a research needs assessment, and the drafting an exploratory grant application (similar to the R21 mechanism of NIH) complete with biosketch and supporting documents.

**FIGURE 2.**
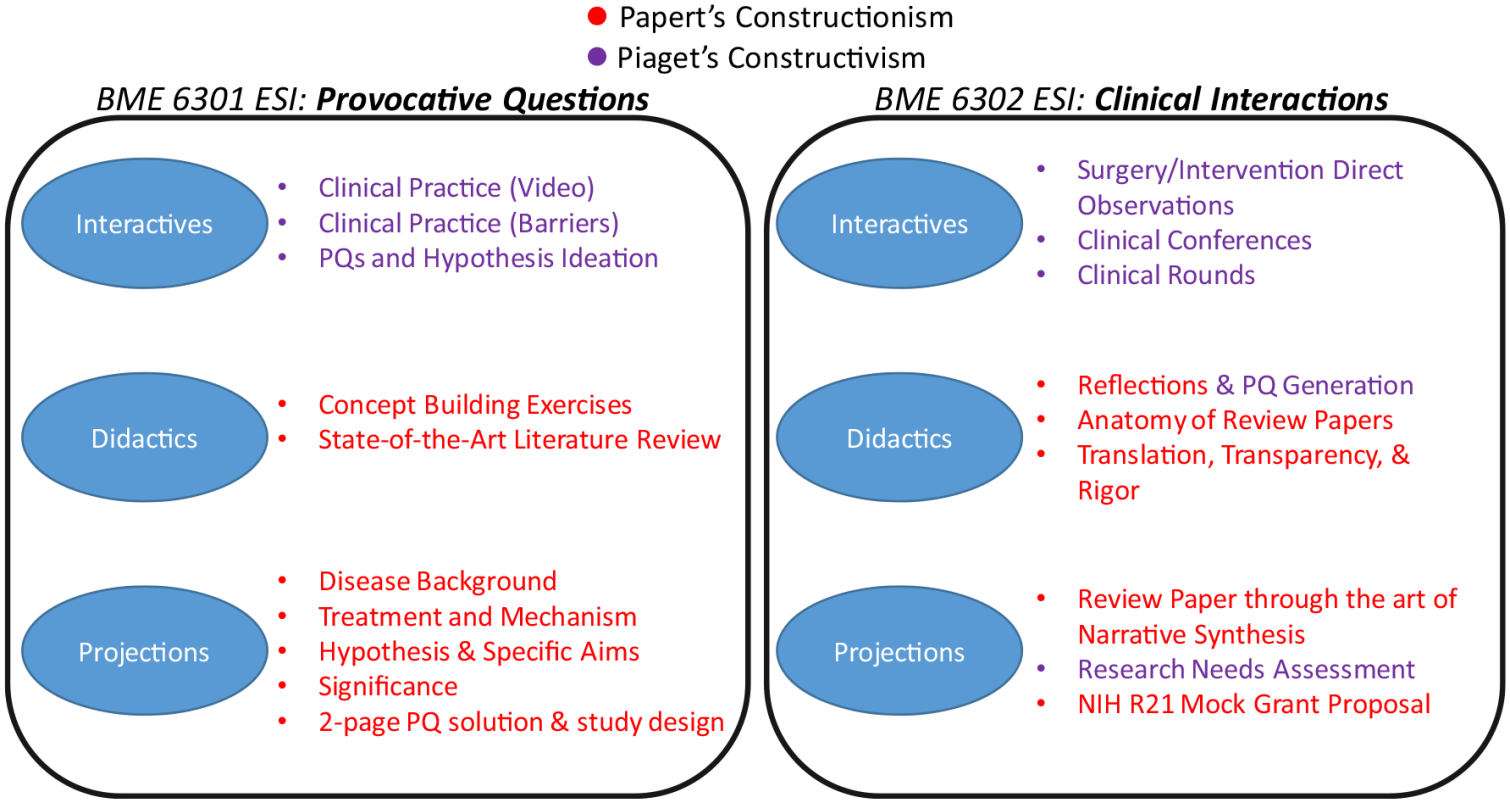
*(left) BME 6301 ESI: Provocative Questions and (right) BME 6302 ESI: Clinical Interactions* activities labeled according to learning approach emphasis.

The clinical immersion core as laid out by *ESI:PQ* and *ESI:CI* represent a series of courses that emphasize both constructionist and constructivist learning approaches. To get a full sense of how the courses work in tandem, Fig. 2 lays out a color-coded (red-constructionism, and purple-constructivism) distribution of the more considerable content according to three categories: (1) Interactives, (2) Didactics, and (3) Projections. As can be seen in Fig. 2-left, *BME 6301 ESI:PQ* has more limited unstructured immersive experiences with Interactives that take place solely in the classroom. The more considerable structured exercises occurring in the form of Didactics and Projections. It should also be noted that the topics listed in the Projections are solely associated with assigned practicals. With respect to *ESI:PQ*, the content aligned with Papert’s constructionist learning model makes up approximately 2/3rds of the course. In *BME 6302 ESI:CI*, the heavier emphasis is on more unstructured learning with time spent in the operating rooms and interventional suites as well as clinical conferences. While Fig. 2-right shows a more substantive emphasis on Piaget’s learning model, it should be further noted that these make up the counterpart scenario to *ESI:PQ*, i.e. approximately 2/3rds of the course focuses on a constructivist approach. Interestingly however, it should be noted that some of the most rewarding and challenging structured Projections are performed in *ESI:CI*, namely, the use of *narrative synthesis*^15^ in the generation of a review paper, and the mock grant proposal.

#### ESI: Technical Core

While the above clinical immersion core is the central feature to the immersion program developed, as noted, the program enrolls students from three possible engineering departments and computer science. Technical skill sets span across these departments to support the domain of *ESI* at Vanderbilt and components from each banded together represent a novel *ESI* core technical curriculum. As a result, understanding the core didactic distribution is an important aspect to understanding the overall *ESI* program. Interestingly, while our design was first initiated at an institutional level in 2015,^16^ others have followed, and similar structures are appearing in surgical and interventional engineering.^17,18^ Briefly, trainees customize their program with technical core courses themed among seven academic thrusts as needed and are described below:

- *Therapy Guidance*, *Delivery*, *and Localization*: Navigation for delivery of therapeutic processes is both technically challenging and medically relevant. By accurately and precisely delivering a therapeutic process—be it drug delivery, surgery, ablation or implantation—both damage to healthy tissue can be reduced, and better therapeutic efficacy to diseased tissue can be ensured. The technical challenges include tracking, registering data spaces to physical spaces, and providing location, distribution, and trajectory data to the physician in a manner that can be easily integrated into their patient treatment plan. Providing the position of the therapeutic device and/or effective treatment zone relative to both diseased and healthy tissue requires a coordinated effort by physician and engineering teams who understand each other’s tasks and vocabulary.
- *Modeling and Simulation for Surgery*/*Intervention*: Modeling and simulation are commonly used for four functions: (1) to understand and provide analysis of data, (2) to provide an educational platform, (3) to predict likely outcomes before costly design, and/or (4) for feedback and control. In *ESI*, modeling and simulation take on new roles which shape therapeutic and surgical processes. For example, the use of a computational model in conjunction with sparse deformation measurements may allow for the accurate volumetric prediction of soft-tissue shift to more accurately target disease. Alternatively, complex models of flexible rods can be used to create all new forms of robotic actuation. Across the domain, modeling and simulation are serving as a constraining scaffold to enable novel developments in guiding and performing therapeutic and surgical/interventional processes.
- *Image Processing*, *Visualization*, *and Analysis*: Over the past two decades, patient imaging information has grown exponentially. Imaging data on physiological, functional, and structural aspects for both diseased/dysfunctional and healthy tissue have direct impact on the delivery of surgical and interventional procedures. Providing timely analysis of such data to physicians for planning/staging, intraoperative guidance, and postoperative care with appropriate presentation frameworks will be essential for safe, effective, and efficient delivery of therapy.
- *Interventional*/*Surgical Therapeutics*: In today’s healthcare, it is not unusual for patients to have many treatment pathways that are highly dependent on a complex myriad of patient-specific factors. Treatment options include novel forms of encapsulated and convective drug release, neurostimulation and modulation, energy-based ablation techniques to name just a few. The options are extensive and the need for systematic understanding within the context of human procedures will be vital in future patient care.
- *Interventional Imaging*: Imaging technology has matured rapidly over the past several decades and the impact of conventional magnetic resonance, computed tomographic, and ultrasound imaging systems on disease diagnostics has been profound. While the direction for imaging science is towards quantitative imaging and biomarker diagnostics, the focus in this area of the training program is toward the effective use of intraoperative imaging techniques to guide and provide feedback to the delivery of surgical and interventional processes. While imaging science advances continue, their translation for use within a highly dynamic and workflow-sensitive environment for procedural medicine is not as well realized. In addition, with more novel automated systems on the horizon, the integration of imaging with devices will be unprecedented. To train our scholars, the fundamentals of imaging instrumentation are re-conceptualized to provide the most pertinent intraoperative feedback or, in the extreme case, to explore comprehensive theranostic devices capable of visualization, delivery, and therapy feedback.
- *Medical Devices and Robotics*: With respect to guiding delivery of therapy, imaging technologies have provided enhanced geometrical understanding and functional information, and, as a result, enhanced localization of tissue domain targets. While clinically compelling, surgical and interventional procedures require interaction with the tissue domain whether it be controlled tissue resection, navigation with minimally invasive approaches, or percutaneous needle-like therapeutic devices. The development and integration of electro-mechanical devices and robotics to enhance and in many cases outperform their human counterpart is an exciting direction for intraoperative platform technologies. These platforms may be even further augmented with the incorporation of technologies that combine sensing and manipulation with the potential of self-navigation and/or advanced frameworks in tele-operation.
- *Interventional and Surgical Data Science*: For several years now, data science in medicine with respect to diagnostics, imaging, epidemiology, and patient electronic health records has made considerable impact on patient care. *ESI* almost by definition produces highly heterogeneous data (both in nature and quality) and acquires that data from arguably one of the most challenging environments—the procedural presentation. With that in mind, a distinct advantage to working in the *ESI* domain is that there is no better moment for characterizing human disease than at the point of surgery and/or intervention. The investment in data science technologies and the harnessing of this data to determine best procedural practices, to predict therapeutic responders, to establish intraoperative factors linked to better (or worse) outcomes, and/or to stratify expertise in establishing training guidelines is a fascinating prospect for research in this technical area.

Lastly, it should be emphasized that the above thrusts are thematic and courses from among the conventional departments that ESI trainees would elect to fulfill their *Technical Core* often blend these themes. For example, both interventional therapeutics, and localization and delivery themes could commonly reside within one 3-credit hour semester course.

#### ESI: Thought Leaders Seminar Series

In addition to the clinical and technical training cores, the *ESI Thought Leaders Seminar Series* is an important contribution exposing trainees to the wider community of current research in the *ESI* domain. In addition to recruiting speakers from around the world, the seminar also serves as a central community building activity bringing faculty and students together to share, discover, and discuss state-of-the-art research as well as ongoing trans-institutional research. The seminar series has several formats which include traditional seminars, informal research discussions, novel dual-speaker talks given by engineer–physician teams, and educational activities (e.g. grant writing workshops, research in progress reports, dissemination of new research techniques, and training in rigor and transparency in research). The seminar series meets approximately biweekly and culminates with an all-day winter symposium that features a keynote from a physician–scientist engaged in translational research within the *ESI* domain. It should also be noted that a summer instructional series is also conducted separate from this that is centered around trainee activities such as Research in Progress seminars (RiPs) and training workshops.

## METHODS

The training program described above is a novel, integrated approach combining engineering knowledge, clinical immersion, and the process of identifying, studying, and investigating the resolution of problems within the *ESI* domain. Given that this program is early in development, data collected on outcomes involves operational metrics and data from self-reported surveys from the trainees. While the more traditional graded didactic components from the *ESI: Technical Cores* are routinely available, for the purpose of this paper, the focus of analysis was on the more novel *ESI: Clinical Immersion Core*. Operational metrics included: (1) the distribution of procedural foci across the different clinical domains in *ESI:PQ*, (2) the number of presentations in each clinical domain as well as the number of practicals adopted in each, (3) the discipline demographic of selected clinical observations in *ESI:CI*, (4) the number of rounds, conferences, surgeries, and different types of surgeries, and (5) the number of clinical contact hours.

With respect to self-reported surveys, five cohorts of students (*n* = 18 trainees) over a 5-year period completed the clinical dual-course core program (note, the fifth cohort has one student still in progress). It should be noted that the survey data was taken after the dual-course suite was completed. Given that the courses are sequential and were conducted over one calendar year for all students, it was determined that a retrospective analysis of the entire clinical core would allow for better introspection and resolution of differences. With respect to each *ESI Clinical Immersion Core*, three common areas were scored by trainees on a scale from 1 to 10 to rate their knowledge levels before and after each course, i.e. *ESI:PQ* and *ESI:CI* (10 being the highest knowledge attainment level). The three common areas that trainees were asked to evaluate before and after the courses were: (1) understanding of procedural medicine, (2) education levels in surgical technologies, and (3) education in translational clinical research. It should be noted that for item (1), *ESI:PQ* trainees were asked about procedural medicine understanding, whereas with *ESI:CI*, trainees were asked about the clinical specialty they elected for their observational experiences. In addition to the above, trainees were asked more comprehensive survey questions regarding the entire core. Among the questions asked, three examples asked trainees to rate: (1) the perceived importance of procedural observation to their research, (2) their ability to pose questions toward important problems in human health, and (3) the perceived breadth of research possible in procedural medicine (note, all of the questions asked are in “Appendix”). With respect to statistical testing, as knowledge of an area would not be anticipated to decrease from before to after taking the core, a standard one-tailed, paired Student’s *t*-test was used to evaluate differences with significance specified as *p* < 0.001 (actual *p*-values are reported in Figs. 5 and 6 captions).^19^ With respect to outlier testing, Grubbs’ test was used.^19^ The collection of data and analysis on the program was approved by the Vanderbilt Institutional Review Board.

## RESULTS

**FIGURE 3.**
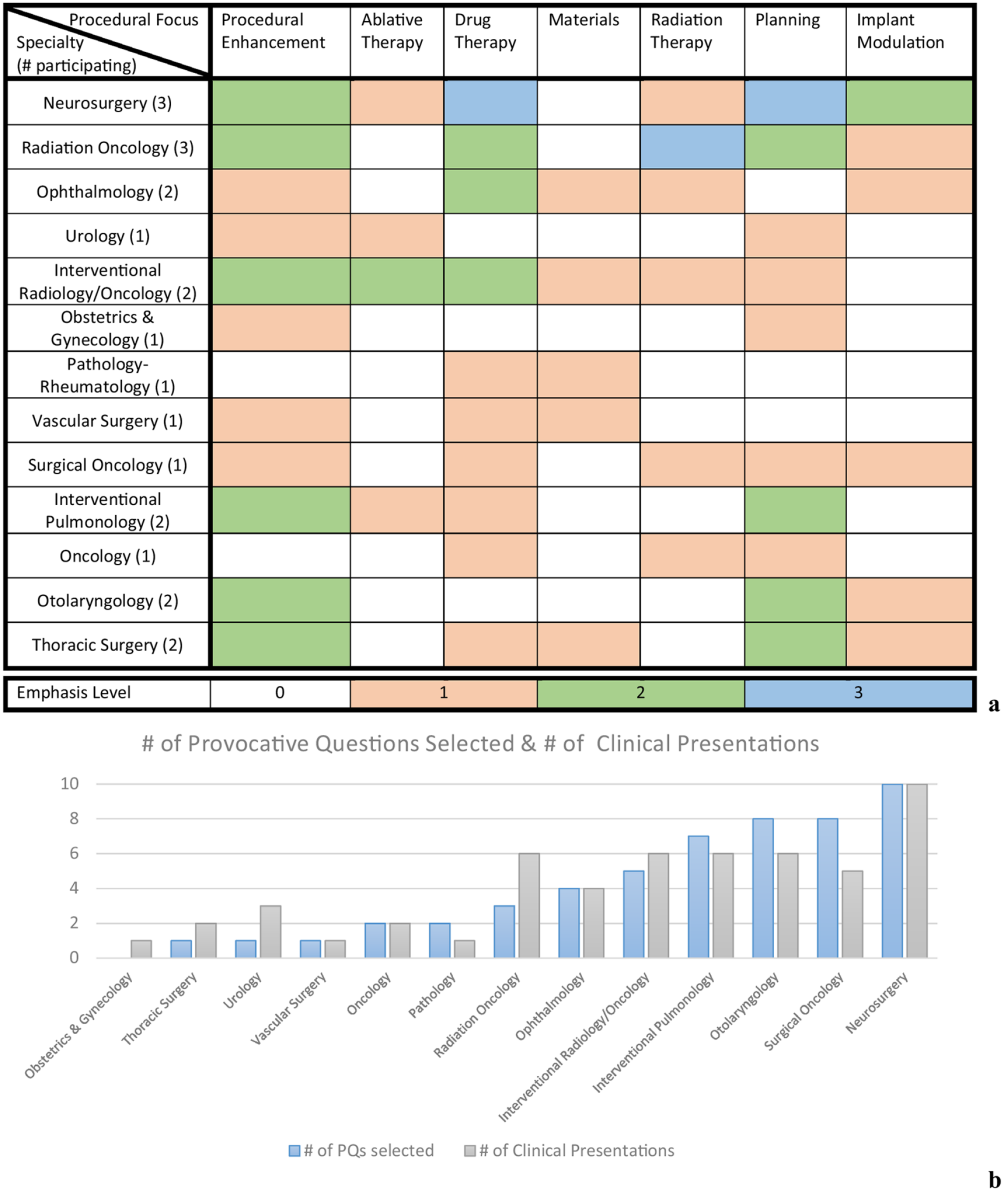
(a) Clinician specialties from *ESI:PQ* and their procedural foci in clinical sessions with emphasis levels provided; and (b) trainee selection of provocative questions domains for practicals and the number of clinical domain presentations over the five cohorts. Note emphasis level reflects the number of separate physician speakers that emphasized coverage of the topic in their talk. For example, all three neurosurgeons that presented in *ESI:PQ* emphasized drug therapy and planning.

Figure 3a is a matrix table illustrating the specialties covered in the 10+ lecture scaffolding of the *ESI:PQ* course over the 5-year offering and the respective emphasis of these lectures with respect to procedural medicine. It is color coded with respect to emphasis and content. For example, with neurosurgery, three different neurosurgeons presented over the time period where all had discussed drug therapies and planning, only one emphasized ablative therapies, and none provided a focus on materials. In Fig. 3a it can also be observed that neurosurgery, radiation oncology, interventional radiology/oncology/pulmonology, ophthalmology, otolaryngology, and thoracic surgery had multiple different speakers during the 5-year period. Observing the matrix in Fig. 3a closely, with respect to the breadth of procedural focus within the clinical scaffolding, many of the specialties discussed procedural enhancement, drug therapeutics, and planning. Figure 3b illustrates a bar chart that provides the selection of provocative questions used for the written practicals of all *n* = 18 trainees. It also shows the number of clinical presentations in each domain over the entire 5 year period. As an example, no students selected provocative questions associated with obstetrics and gynecology which was only presented in 1 year while many students pursued provocative questions in the areas of neurosurgery and it was taught every year by two surgeons. On this last example, each year of *ESI:PQ* had one surgeon present on brain tumor surgery, while the second presented on functional neurosurgery. While functional neurosurgery is a subspecialty, the questions and concerns within the two areas are quite different.

With respect to selected trainee clinical observations in the second immersive course, *ESI:CI* involved 16 different clinicians reflecting 11 different specialties: otolaryngology, pathology, orthopedic surgery, surgical oncology, interventional cardiology, obstetrics/gynecology, interventional oncology, ophthalmology, interventional pulmonology, hepatobiliary surgery, and neurosurgery. While 7 of the 11 specialties only had one trainee, neurosurgery, interventional pulmonology, ophthalmology, interventional oncology, and orthopedic surgery had multiple trainees select observational experiences. To give some sense of trainee experiences reported in the *Reflections* activity of *ESI:CI*, the following were reported: (1) a complete organ harvest for transplant as well as open liver surgery, (2) the integration of ultrasound imaging, catheter-based navigation, selection of device, and completion of a transcatheter aortic valve replacement, (3) a series of cryo-biopsies in the lung, and (4) brain tumor resections in pediatric patients.

**FIGURE 4.**
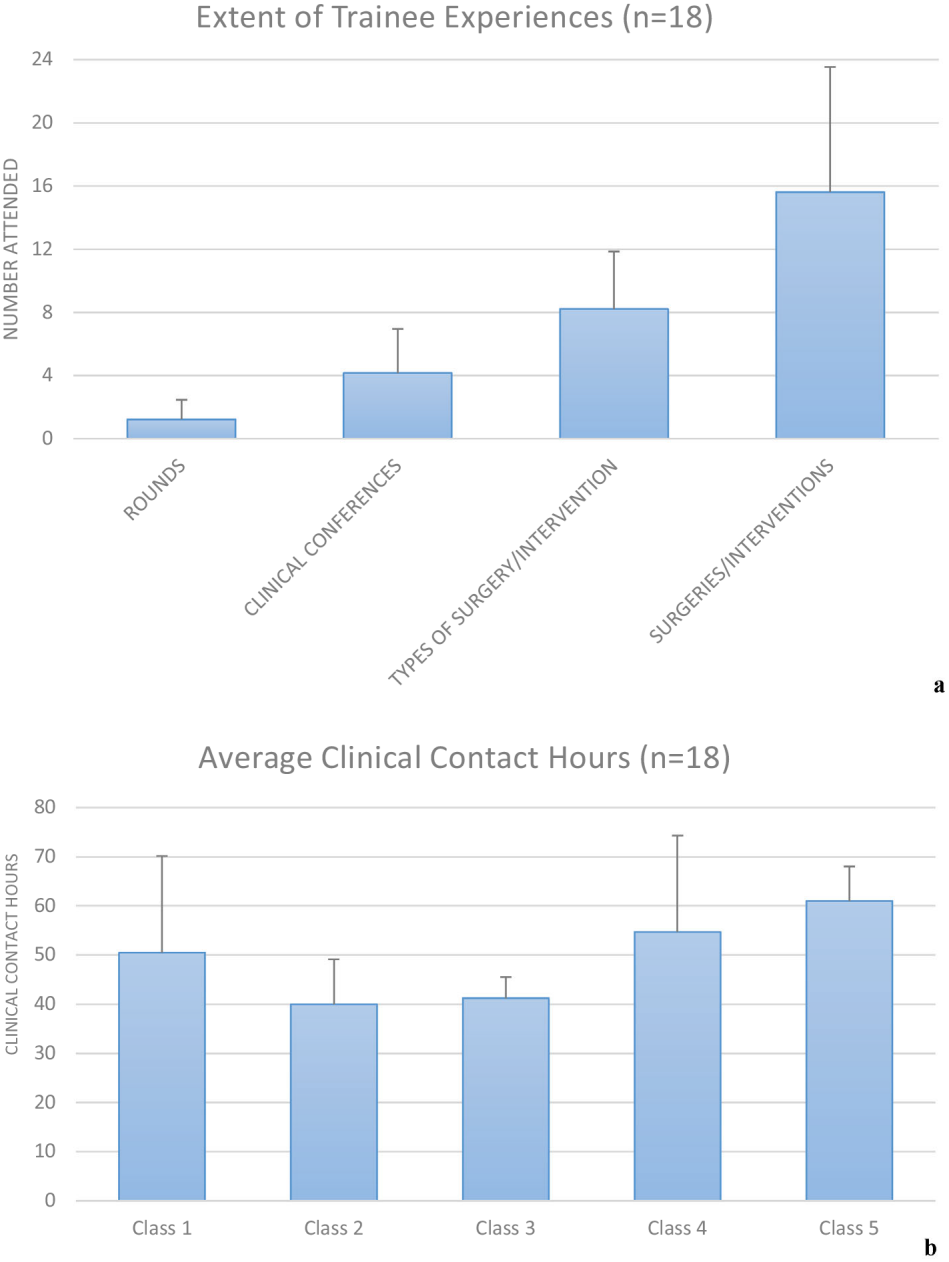
(a) Extent of trainee experiences; and (b) number of clinical contact hours per trainee class.

Figure 4a quantifies the types of clinical interactions associated with *ESI:CI* while Fig. 4b quantifies the average number of clinical contacts hours associated with each year’s cohort among the *n* = 18 trainees. Briefly, trainees experienced an average of 15.6 ± 7.9 surgery/intervention observations with an average of over 8.2 ± 3.6 different types of surgeries/interventions among those for each student. The trainees experienced an average of 4.2 ± 2.8, and 1.2 ± 1.3 clinical conferences, and clinical rounds, respectively. With respect to clinical contact hours, the average clinical contact time across the cohort was 48.2 ± 14.7 h with a median of 42 h. It should be noted in analyzing the data associated with Fig. 4a, an outlier was detected with respect to the number of surgery and interventions. One student who could watch repeated minor orthopedic surgeries could see several per day and observed a total of 50 procedures. When removing this outlier, trainees still experienced an average of over 13 surgeries/interventions with a median of 12 surgeries/interventions.

**FIGURE 5.**
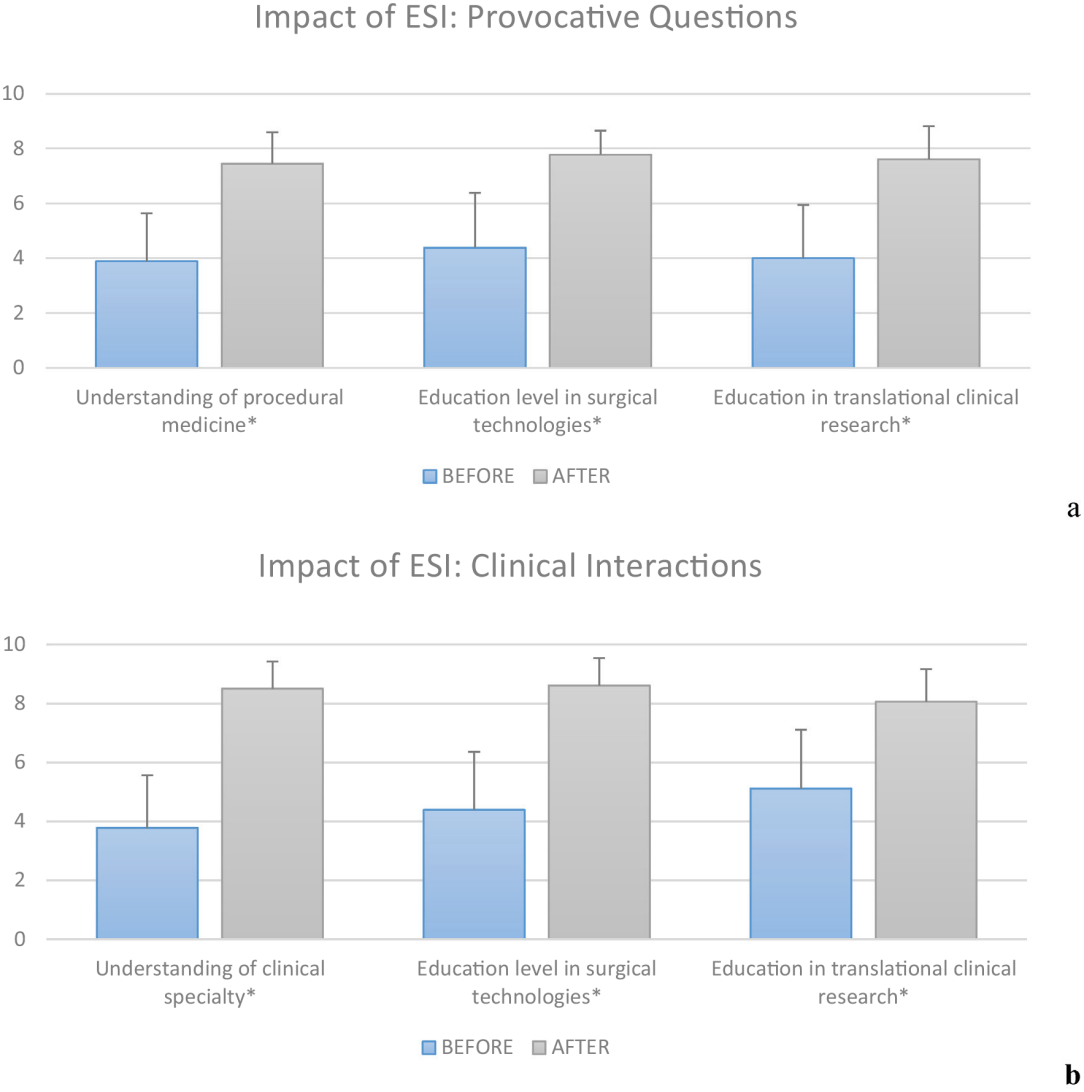
Assessed impact (a) impact before and after *ESI:PQ*; and (b) before and after *ESI:CI*. *Statistical significance comparing before to after with (a) *p* = 8e–10, 8e–7, 7e–8, respectively; and (b) *p* = 5e–10, 3e–8, 9e–7. With respect to vertical scale—1: represents no attributable value, and 10: represents high attributable value of each category.

Figures 5a and 5b relates the impact of each *ESI* core course before and after completion as self-reported by *n* = 18 trainees with respect to improving trainee understanding of (1) procedural medicine, (2) surgical technologies, and (3) translational clinical research. It should be noted that all conditions shown in Figs. 5a and 5b demonstrated a statistically significant increase in perceived knowledge base (*p* < 0.001). With respect to understanding of procedural medicine and specific specialty, *ESI:PQ* and *ESI:CI* reported remarkably similar 10-point baseline averages scores of 3.9 ± 1.7, and 3.8 ± 1.8, respectively. The perception of knowledge improvement in procedural medicine and specialty at the conclusion of each course yielded an average score of 7.4 ± 1.1 (35% increase) and 8.5 ± 0.9 (47% increase), respectively. With respect to understanding surgical technologies for both courses, both *ESI:PQ* and *ESI:CI* reported an average 10-point baseline score of 4.4 ± 2.0. The perception of knowledge improvement in understanding surgical technologies at the conclusion of each course yielded an average score of 7.8 ± 0.9 (34% increase) and 8.6 ± 0.9 (42% increase), respectively. These represent a considerable impact with statistically-significant improvement on average (*p* < 0.001). It should also be noted that the average 10-point score after *ESI:CI* when compared to the average 10-point score after *ESI:PQ* was statistically better for both categories of knowledge, i.e. procedure/specialty, and surgical technology (*p* = 0.0009 concerning procedural medicine, and *p* = 0.0003 concerning surgical technologies). When comparing the perceived knowledge in these categories prior to taking the courses, there was no statistical difference in the average 10-point baseline scores (*p* = 0.41 concerning procedural medicine, *p* = 0.5 concerning surgical technologies). Lastly, for every category above listed in Figs. 5a and 5b (knowledge of procedural medicine/specialty, surgical technologies, and translational clinical research), every individual trainee (*n* = 18) registered an increase in knowledge in every category, i.e. no trainee reported a decrease or stagnation in their understanding.

**FIGURE 6.**
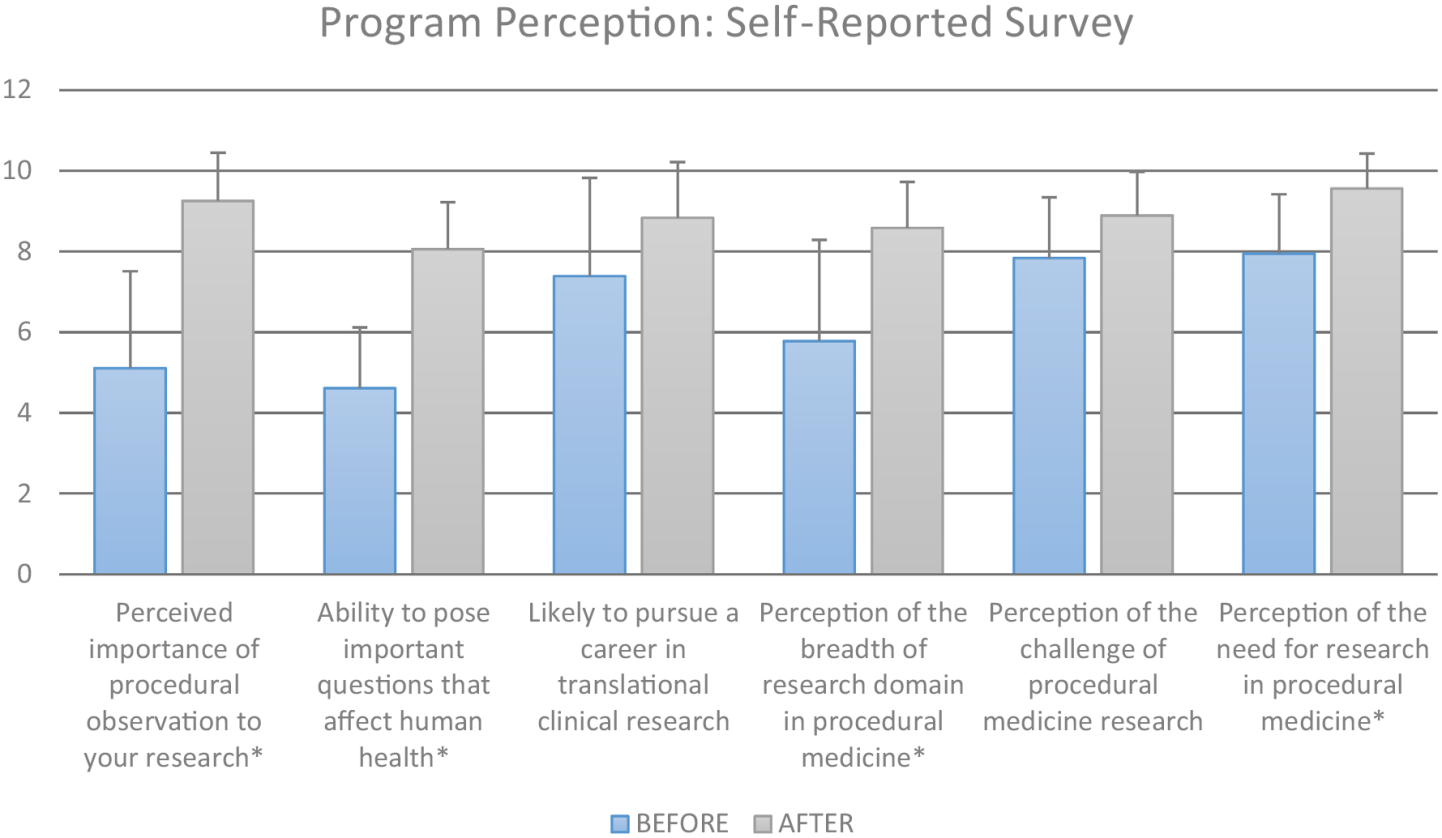
Trainee perception of overall program impact and goals. *Statistical significance comparing scores before to those after with *p* = 4e–7, 2e–9, 0.004, 2e–5, 0.009, 2e–4, respectively. With respect to vertical scale—1: represents no attributable value, and 10: represents high attributable value of each category.

Figure 6 evaluates the impact of the entire dual-course clinical core with respect to the trainees evaluating the importance of procedural observations to their own research, their abilities to pose important questions affecting human health, and their perception of research breadth within procedural medicine among others. It should be noted that all conditions in Fig. 6 demonstrated a statistically significant increase (*p* < 0.001) except for the already highly scored questions associated with pursuing a career in translational clinical research and the trainees’ perception of the challenge of procedural medicine research. With respect to the perceived importance of observation for their research, the baseline 10-point score average across trainees was 5.1 ± 2.4 with an increase following the program to 9.3 ± 1.2, an approximate 42% improvement. With respect to trainees evaluating their ability to pose important questions that affect human health, trainees had an average baseline 10-point score of 4.6 ± 1.5 which statistically improved to 8.1 ± 1.2 (*p* < 0.001), an improvement of 35% after taking the training core. Lastly, similar to Fig. 5, for both categories (importance of procedural observation, and ability to pose questions that affect human health), every individual trainee (*n* = 18) registered an increase in knowledge with no trainee reporting a decrease or stagnation in their understanding.

## DISCUSSION

One of the hallmarks of the program reported herein is the early exposure to procedural medicine. With *ESI:PQ*, trainees received an exposure to a wide variety of procedural medicine typically within their first year of graduate study. Within this course, physicians conveyed the nature of disease/dysfunction, some typical presentations of patients, the mechanics and instrumentation involved with therapy, and the workflow of procedures in which they are involved. They often would relay the spectrum of outcomes, as well as the challenges and barriers faced in the delivery of procedural care. The physician would then typically codify these experiences and observations into the form of provocative questions for the trainees. These clinical sessions provided enormous value in grounding the trainees in procedural medicine but also demonstrated a wide range of procedural foci heterogeneity across different specialties. Some common themes certainly emerge across specialties with presentations focusing on technologies to enhance procedures, the impact of drug therapies, and the value in planning with 75+% of the specialties having at least some level of emphasis in these areas. Broadly speaking in the selection of provocative question topics by trainees, the areas of interventional pulmonology, otolaryngology, surgical oncology, and neurosurgery all have elevated topic selection versus the number of presentations in the areas across the 5-year period. It should be noted that there are a considerable number of research projects among these clinical domains.

Moving on to assessing clinical cadre engagement over the 5-year period, in the first year of the program, the *ESI:PQ* course began with 10 participating physicians. At the conclusion of the fifth year, there are a total of 22 speakers available for rotation in the *ESI:PQ* course. This reflects an average change of approximately 24% per year, or 2–3 new speakers being enlisted to present each year. This demonstrates a substantive clinical interest from our physician cadre in working with graduate student engineers.

When considering trainee experiences, the data reflects trainees not only being able to see many procedures but also many different types of procedures. Among existing immersion programs for graduate engineering students, to our knowledge, none have such an extensive and concentrated clinical contact structure. From its inception, *ESI:PQ* was to serve the purpose of providing exposure to a variety of clinical domains. It was designed to allow students to see both procedural differences and commonalities across specialties. More specifically, within the context of interim non-clinical lectures and looking across clinical presentations, students are encouraged to link and reflect on the diagnostic data driving procedural decisions, the biophysics of treatment, the instrumentation and techniques employed, the clinical goals desired, the outcomes and metrics resulting from treatment, and finally the complications ensuing from suboptimal success. With that exposure completed, trainees move on to concentrate on a single specialty rather than a rotation framework like other programs have instituted. The reality is that surgical and interventional disciplines are complex. Their mastery requires detailed understanding of disease/dysfunction and therapeutic response, while also needing equal understanding of therapy delivery and controlled technique. Surgeons and interventionalists spend a career learning and developing techniques to integrate those goals. In recognition of that complexity, *ESI:CI* was designed to provide a focused yet extensive observation experience in a clinical specialty. This emphasis prevents trainees from becoming overly fixated on any one aspect of a procedure and facilitates a deeper understanding of the wider treatment domain and workflow to potentially provide a wider impact of their research in the field. Trainee experiences also report an average attendance of 4+ clinical conferences (e.g. hepatocellular carcinoma—HCC—conference, functional neurosurgery conference, or neurosurgery tumor board, *etc*.). It is in these conferences where the trajectory of care is often observed directly where multiple specialties come together to consider the comprehensive care of a patient. For example, the HCC conference often has hepatobiliary surgeons, radiologists, interventional oncologists, and oncologists discussing cases. Trainees had the opportunity to observe such interactions with their clinical mentor, an invaluable experience. With respect to the clinical contact hours, the average contact hours reported was considerably larger than the median. In analysis, it was found that the difference between mean and median represented a positive skewing of the data. Looking at the individual reports, the range of contact time was between 30 and 80.5 hours for trainees with two trainees having 80+ h of clinical contact time. Lastly, it should be noted that trainees attended a very limited number of clinical rounds, an average of just over one experience. As the point of the program is to observe surgical and interventional physical procedural aspects, this limited number of rounds was by design. While still welcome, the course instructor used this metric as a means to assess if a student was not receiving the intended training. In this case, a small number of clinical round observations was seen as meeting the goal of the program.

While the self-perceived improvement in each of the categories for *ESI:PQ* and *ESI:CI* is considerable, the finding that there was a statistical difference between the post-course scores comparing *ESI:PQ* to *ESI:CI* in the categories of understanding the procedural specialties and surgical technologies was rewarding as well. This may suggest that trainees perceived their understanding of procedural medicine and surgical technologies to be enhanced by the clinical immersion experience of *ESI:CI* when they physically spent time in the operating room observing versus the scaffold lecture design of *ESI:PQ*. It would be interesting to get a better understanding of what comprises that difference. As we look at just raw score performance, we see an 8–12% increase in scores but it is difficult to know if the same comparative factors are embedded in these differences. For example, given that *ESI:PQ* and *ESI:CI* are designed favoring a broad constrained didactic presentation in the former, and an intense environmentally-immersive clinical experience in the latter, the significance could simply reflect those differences in experiential nature and not necessarily increased understanding. Future work must involve surveys with better discriminating ability.

Considering that a great deal of this curriculum design is uniquely focused at generating provocative questions and hypothesis generation, the data presented are quite satisfying. In particular, the strong responses in the importance of observation and the ability to pose important questions that affect human health, are noteworthy. While this is clearly a biased group, such strong responses in a post-program review are quite encouraging.

Lastly, the results and discussion above certainly have limitations. This is a self-identifying, self-reporting population that is already quite focused toward research endeavors aimed at clinical translation. This is suggested by the question regarding the likelihood that the trainee will pursue translational clinical research with the trainees having an average score of 7.4 ± 2.4 before participating in the dual-course program. Similarly, when trainees were asked to rate the perceived challenge of translational work, the challenge is rated quite high at an average of 7.8 ± 1.5 prior to the program. When considering the changes in these scores after participation, while both increased, neither were found to be statistically significant (*p* = 0.004, 0.009, respectively). Interestingly, with respect to the individual trainee ratings of likelihood to pursue translational research, and the perceived challenge of translational work, approximately 17% (*n* = 3), and 39% (*n* = 7) had 10-point scores that did not change, respectively. The above characteristics certainly suggest a population that strongly self-identifies with clinical translational research. It is difficult to factor out such bias in that it would require participation in the program from those not expressing interest in procedural care within their training. Another shortcoming of the work is that trainee outcomes upon completion of their doctoral work are not reported yet. Some interim findings are known with approximately 39% of trainees receiving a competitive extramural fellowship award of some form (either pre- or post-doctoral). With respect to trainees moving on, only four trainees have finished their doctoral work with two taking post-doctoral positions (one received a prestigious post-doctoral fellowship) and the remaining two moving on to industrial positions (a startup and a large medical device/imaging company). Another limitation, the survey design was not a particularly sophisticated one. Additional questions to provide a more discerning understanding of the findings are warranted. Perhaps a more detailed rubric-based analysis of the assignments collected (practicals and proposal writing) across training years will yield important findings. Similarly, it would be interesting to identify matched pre-doctoral students who are involved with surgical and interventional research and who are not enrolled in the dual-course suite and perhaps compare final doctoral thesis contributions with an objective rubric. Lastly, with a retrospective survey of the entire program and course suite happening at the end of the program, perhaps responses are somewhat repetitive. While possible, the statistical difference between the after course 10-point scores between *ESI:PQ* and *ESI:CI*, i.e. the 8–12% increase noted above, would suggest that the trainees are discerning a different value. Granted the survey tool in this case was relatively blunt, but the results would suggest support for the learning approach.

## CONCLUSIONS

This paper reports on a novel pre-doctoral training program focused on the role of engineering within the fields of surgery and intervention. Perceived importance and knowledge gained from the program, while self-reported, suggest substantive experiential learning is taking place. With respect to future work, clearly long-term outcomes need to be analyzed. While traditional metrics such as publications, awarded grants, career awards and honors, or placement of future trainees are important, there are other tangible outcomes that would be fascinating to consider. Perhaps the heavy emphasis on bridging technologies and procedural medicine will result in career path predilections? It would be interesting to know whether investigators truly take on roles at the interface of technology and procedural medicine. Similarly, will trainees create technologies that more readily translate? Perhaps, a metric will be how many patients’ care have been affected by their efforts? Or how many human-based system research studies they have performed? And/or how many patients enrolled in studies they directed? It would also be interesting to survey the impact of work by trainees that led to important findings discovered in the presentation and treatment of human disease and measured by their human systems research designs. Given the working domain of procedural medicine, and the focus on translational research, the potential for rewarding outcome studies is exciting to consider. Clearly, future work must include more objective metrics and documented outcomes. However, the initial experience presented here certainly portends of potentially paradigm-shifting training for engineering pre-doctoral students in the fields of surgery and intervention.

## Data Availability

Data is available.

## APPENDIX

The below is a survey of 31 questions used as a tool to understand the value of the dual-course core as part of our training program. Questions 4–31 used a 1–10 point scale to assess.

### SURVEY

1. What department are you pursuing your PhD in?
2. Are you an MD/PhD student?
3. How many years were you a graduate student at Vanderbilt prior to your enrollment in the Provocative Questions course?

Provocative Questions Course

1. How educated did you feel about the areas of procedural medicine associated with surgery and intervention before enrolling in the Provocative Questions Course? (1-not educated, 10-highly educated)
2. How educated did you feel about the areas of procedural medicine associated with surgery and intervention after completing the Provocative Questions Course? (1-not educated, 10-highly educated)
3. In the Provocative Questions Course, 10 or more different clinicians provided lectures on their areas of expertise. Did you feel this covered a breadth of procedural medicine associated with surgery and intervention? (1-no breadth, 10-high breadth)
4. Prior to the Provocative Questions Course, how educated were you in the technologies associated with performing procedures in surgery and intervention? (1-not educated, 10-highly educated)
5. After completing the Provocative Questions Course, how educated were you in the technologies associated with performing procedures in surgery and intervention? (1-not educated, 10-highly educated)
6. Prior to the Provocative Questions Course, how educated were you in the area of translational clinical research in surgery and intervention? (1-not educated, 10-highly educated)
7. After the Provocative Questions Course, how educated were you in the area of translational clinical research in surgery and intervention? (1-not educated, 10-highly educated)

Clinical Interactions Course

1. Prior to the Clinical Interactions Course, how educated did you feel in the clinical specialty you were observing? (1-not educated, 10-highly educated)
2. After the Clinical Interactions Course, how educated did you feel in the clinical specialty you had observed? (1-not educated, 10-highly educated)
3. After the Clinical Interactions Course, did your perception of the clinical specialty change? (1-no change, 10-fundamental conceptual change)
4. Prior to Clinical Interactions Course, how educated were you in the technologies associated with performing procedures in that clinical specialty? (1-not educated, 10-highly educated)
5. After Clinical Interactions Course, how educated were you in the technologies associated with performing procedures in that specialty? (1-not educated, 10-highly educated)
6. Prior to the Clinical Interactions Course, how educated were you in the area of translational clinical research in surgery and intervention? (1-not educated, 10-highly educated)
7. After the Clinical Interactions Course, how educated were you in the area of translational clinical research in surgery and intervention? (1-not educated, 10-highly educated)

General Questions on Core

1. Prior to enrolling in the T32 Training Core Courses of Provocative Questions and Clinical Interactions, how important did you believe procedural observation was to your research? (1-not important, 10-highly relevant)
2. Upon Completion of the T32 Training Core Courses of Provocative Questions and Clinical Interactions, how important do you believe procedural observation is to your research? (1-not important, 10-highly relevant)
3. Prior to enrolling in the T32 Training Core Courses of Provocative Questions and Clinical Interactions, how capable were you in posing research questions that had high value for human health? (1 – not capable, 10-highly capable)
4. After completing the T32 Training Core Courses of Provocative Questions and Clinical Interactions, how capable are you in posing research questions that have high value for human health? (1-not capable, 10-highly capable)
5. Prior to enrolling in the T32 Training Core Courses of Provocative Questions and Clinical Interactions, how likely were you to pursue a career in translational clinical research? (1-will not pursue translational research, 10-highly likely to pursue translational research)
6. After completing the T32 Training Core Courses of Provocative Questions and Clinical Interactions, how likely are you to pursue a career in translational clinical research? (1-will not pursue translational research, 10-highly likely to pursue translational research)
7. Prior to enrolling in the T32 Training Core Courses of Provocative Questions and Clinical Interactions, how diverse a research domain was your perception of procedural medicine, in particular surgery and intervention? (1-not diverse, 10-very diverse)
8. After completing the T32 Training Core Courses of Provocative Questions and Clinical Interactions, how diverse a research domain was your perception of procedural medicine, in particular surgery and intervention? (1-not diverse, 10-very diverse)
9. Prior to enrolling in the T32 Training Core Courses of Provocative Questions and Clinical Interactions, how challenging did you believe research was in the area of procedural medicine, in particular surgery and intervention? (1-not difficult, 10-highly difficult)
10. After completing the T32 Training Core Courses of Provocative Questions and Clinical Interactions, how challenging do you believe research is in the area of procedural medicine, in particular surgery and intervention? (1-not difficult, 10-highly difficult)
11. Prior to enrolling in the T32 Training Core Courses of Provocative Questions and Clinical Interactions, how necessary did you believe research was needed in the area of procedural medicine, in particular surgery and intervention? (1-not difficult, 10-highly difficult)
12. After completing the T32 Training Core Courses of Provocative Questions and Clinical Interactions, how necessary do you now believe research is needed in the area of procedural medicine, in particular surgery and intervention? (1-not difficult, 10-highly difficult)
13. In the era of healthcare reform, how important is research in procedural medicine, in particular surgery and intervention? (1-not important, 10-critically important)
14. In the era of healthcare reform, what do you perceive is the future of research in in procedural medicine, in particular surgery and intervention? (1-bleak, 10-bright future)
